# Relationship between occupational noise exposure and hypertension: Cross-sectional evidence from real-world

**DOI:** 10.3389/fpubh.2022.1037246

**Published:** 2022-12-12

**Authors:** Ling Zhang, Siqi Chen, Zhuowang Chen, Wenjun Yin, Wenjuan Fu, Fang He, Zhen Pan, Guilin Yi, Xiaodong Tan

**Affiliations:** ^1^Department of Preventive Medicine, School of Public Health, Wuhan University, Wuhan, China; ^2^Wuhan Prevention and Treatment Center for Occupational Diseases, Wuhan, China

**Keywords:** occupational health, occupational noise, hearing loss, hypertension, nomogram

## Abstract

**Background:**

Occupational noise is one of the most common and prevalent occupational hazards worldwide and may induce adverse auditory and/or non-auditory health effects. However, the relationship between occupational noise exposure and hypertension is controversial and has long been debated.

**Methods:**

Based on large sample cross-sectional data from all registered occupational health examination units from 2021 to 2022 (*N* = 101,605), this study aimed to analyze the prevalence of hearing loss and hypertension and to explore the influencing factors of hypertension of workers in Wuhan. Descriptive statistics, univariate analyses and multivariate analyses were used. Forest plot and nomograms were constructed for the visualization of predictive results. The ROC curve, AUC, C-index and calibration curves were used to assess the predictive accuracy and validity. DCA was performed to evaluate the net benefit that workers could receive.

**Results:**

Higher rate of high-frequency hearing loss (25.3%), speech frequency hearing loss (8.8%), ECG abnormalities (31.9%) and hypertension (21.0%) were found in workers exposed to occupational noise in Wuhan. Occupational noise exposure (OR = 1.09, 95% CI: 1.01–1.18, *p* = 0.04), growth of age (OR: 1.07, 95% CI: 1.07–1.07, *p* < 0.001), overweight (OR: 1.82, 95% CI: 1.73–1.92, *p* < 0.001), obesity (OR: 3.62, 95% CI: 3.42–3.83, *p* < 0.001), hyperglycemia (OR: 1.84, 95% CI: 1.73–1.96, *p* < 0.001), hypercholesterolemia (OR = 1.34; 95% CI 1.22–1.48; *p* < 0.001), ECG abnormalities (OR = 1.11; 95% CI 1.07–1.15; *p* < 0.001) and family history of hypertension (OR = 1.69; 95% CI 1.58–1.81; *p* < 0.001) were risk factors of hypertension for workers. Male workers had a relatively higher hypertension risk than female workers (OR = 1.61; 95% CI 1.54–1.69; *p* < 0.001). Ear protective measures could not reduce the risk of hypertension in workers. Our nomogram has good predictive accuracy and validity. A dynamic nomogram to predict the workers' risk of hypertension was established publicly available online.

**Conclusion:**

Occupational noise exposure may elevate workers' hypertension risk. More effective and relevant prevention measures should be taken. Our nomogram may help identify high-risk workers and facilitate timely interventions.

## Introduction

Hypertension (ICD10 I10-I15) is a common chronic non-communicable disease and the main risk factor of cardiovascular and cerebrovascular diseases (1, 2). Besides high disability and high mortality, it may also cause a heavy burden to the patient, family and society (3). According to Global Burden of Disease Study 2019 (GBD 2019), there were about 10.85 million deaths caused by hypertension worldwide in 2019, accounting for 31% of all causes of death (4). From 1990 to 2019, the deaths induced by hypertension among Chinese residents increased from 1.2 to 2.6 million (5). Apart from socioeconomic and personal factors (such as dietary and exercise habits) (6–8), incidence of hypertension might be influenced by environmental risks such as air pollution and noise (9–13).

Occupational noise is one of the most common and prevalent occupational hazards of the modern world and health effects of noise were first recognized in occupational settings (14). With the development of modern industrialization, occupational noise exposure related to occupational injury has gradually attracted public attention. It has long been established that occupational noise exposure may induce adverse auditory health effects (14–16) and non-auditory health effects (17–20), while the effect of occupational noise exposure on hypertension has been controversial. Existing studies report conflicting results on it (21–23). A prospective cohort study shows that workers exposed to noise levels between 82 and 106 dB for 3–17 years may increase the risk of hypertension with a non-linear exposure-response pattern (22). A landmark finding from animal studies was the demonstration that a chronic exposure to noise with average sound pressure level (SPL) of 85 dB may elicit sustained elevations in monkeys' blood pressure by 30 mmHg without a return to baseline values after the noise ended (24). Some systematic review studies support the association between occupational noise exposure and hypertension (23, 25, 26), while a systematic review with meta-analysis from the WHO/ILO Joint suggests that there is insufficient evidence on the burden of cardiovascular disease (CVD) attributable to occupational exposure to noise appears (21). Yet this research was quickly criticized and questioned by peers (27). Although the authors have responded to the doubts (28), it may not be that much convincing. There is still uncertainty about whether hypertension is associated with occupational noise exposure to date.

As data beyond traditional clinical trials, real-world data (RWD) come from the real medical environment and may reflect the health status of the population under real conditions. According to the latest definition from US Food and Drug Administration (FDA) (29), RWD are data relating to patient health status and/or the delivery of healthcare routinely collected from a variety of sources. RWD play an increasing important role in healthcare decisions (30) while many of them are not sufficiently used, such as physical data from physical examination organizations, leading to data waste and the efficiency of physical examination. Medical records of regular occupational health examinations is such RWD. To prevent occupational diseases, Chinese government require enterprises engage third-party organizations to identify and detect occupational hazards to which workers may be exposed. Workers identified with occupational hazards exposure would attend regular occupational health examinations.

Using occupational health examination data from all registered occupational health examination units (37 units) in Wuhan from 2021 to 2022, the objectives of this study were: (1) estimate the current prevalence of hearing loss and hypertension in workers with occupational risk exposure in Wuhan; (2) examine whether there is potential association between hypertension and occupational noise exposure.

## Materials and methods

### Study population and data source

Our study population were workers with occupational hazards exposure in Wuhan. Data used in this study were medical records of regular occupational health examinations from all registered occupational health examination units (37 units) in Wuhan from January 2021 to May 2022. One lakh six thousand nine hundred thirty-one workers aged 18–60 years exposed to different occupational hazards from 1,264 enterprises in Wuhan, Hubei were enrolled.

Data used in this study comprised of two parts: questionnaire survey and physical examination data. Questionnaire survey contained the workers' demographic information, sociological information and medical history. The demographic information module included workers' sex, age, conscious symptoms and their occupational history (e.g., unit, occupational hazard factors exposed and the duration of exposure). The sociological information module mainly investigated the disease history of the respondents' immediate relatives (parents, grandparents). The medical history module included the type of disease, time, treatment process, disease outcome and so on. Based on the technical specifications for occupational health monitoring GBZ188-2014 (31), physical examination data included height, weight, blood pressure, pulse, blood routine, urine routine, blood lipids, blood glucose, electrocardiogram (ECG), liver function and audiologic testing. All physical examinations were performed by occupational health physicians from 37 registered occupational health examination units in Wuhan. Each worker had the right to refuse participation in health examinations or questionnaire survey partially or completely.

### Definition and assessment of variables

Blood pressure was measured after 5 min of rest in a quiet area. Hypertension was defined as systolic blood pressure (SBP) ≥ 140 mm Hg and/or diastolic blood pressure (DBP) ≥ 90 mm Hg, or antihypertensive medication use (3).

The mean body mass index (BMI) was computed by the ratio of body weight (kg) to height squared (m^2^). Overweight (including obesity) was defined as BMI ≥ 24, normal as 18.5 ≤ BMI < 24 and underweight as BMI < 18.5 using the Working Group on Obesity in China criteria (32). Obesity as defined as BMI ≥27 in this study.

The normal level range of Chinese fasting blood glucose (FBG) is 3.9–6.0 mmol/L (33). Hyperglycemia was defined as FBG > 6.0 mmol/L and hypoglycaemia was defined as FBG < 3.9 mmol/L. Hypercholesterolemia was defined as total cholesterol(TC) ≥ 6.2 mmol/ L (34). The blood samples were collected in the morning on an empty stomach.

Pure-tone audiometry was used to measure the workers' thresholds of hearing at 0.5, 1, 2, 3, 4 and 6 kHz on the basis of diagnostic criteria GBZ 49-2014 (35). Only workers at positions exposed to noise would participate in audiometric examinations. The symbols *HL*_500*Hz*_, *HL*_1000*Hz*_, *HL*_2000*Hz*_, *HL*_3000*Hz*_, *HL*_4000*Hz*_, *HL*_6000*Hz*_ represented the listener's hearing level threshold at a particular pure-tone frequency, in decibels (dB). The subscript L stood for the left ear and L for the right. The binaural high frequency threshold average (BHFTA) and monaural threshold of weighted value (MTMV) were calculated using formula (1) and formula (2), the units were dB. It was defined as high-frequency hearing loss when BHFTA exceeded 25 dB, and normal high-frequency hearing function when BHFTA was < 25 dB. The lower MTMV value of two ears was labeled as *MTMV*_*better*_. It was defined as speech frequency hearing loss when *MTMV*_*better*_ exceeded 25 dB.


(1) $$\begin{array}{l} BHFTA=\\ \frac{HL_{L3000Hz}+HL_{L4000Hz}{+HL_{L6000Hz}+HL_{R3000Hz}+HL_{R4000Hz}+HL}_{R6000Hz}}{6} \end{array}$$

(2) $$\begin{array}{l} MTMV=\frac{HL_{500Hz}+HL_{1000Hz}+HL_{2000Hz}}{3}\times 0.9\\ \;+HL_{4000Hz}\times 0.1 \end{array}$$

### Ethical considerations

Ethical approval was granted by the ethics committee of Wuhan Prevention and Treatment Center for Occupational Diseases (approval number 2022-WZF03).

### Statistical analyses

Data analysis and visualization were performed using R statistical software (version 4.2.1). Group differences of continuous data were analyzed by Student's *t*-test and categorical data by chi-square test (α = 0.05). Multivariate analysis was performed using binary logistic regression. Forest plot (36) and nomograms (37) were constructed for the visualization of statistical predictive models based on binary logistic regression model. The receiver operating characteristic (ROC) curve, area under the ROC curve (AUC), concordance indexes (C-index) and calibration curves were used to assess the predictive accuracy, the discrimination and calibration of the nomogram and internally validated. Decision curve analysis (DCA) was performed to evaluate the net benefit that workers could receive. Internal validation and external validation were carried out, respectively. The bilatera *P* < 0.05 was considered to be statistically significant.

## Results

### Descriptive characteristics of wokers

After elimination of respondents who had absence of blood pressure records (*n* = 2,690), occupational history (*n* = 421), or had hypertension before work (*n* = 2,215), 101,605 respondents were finally included in our analysis. The mean age of the respondents was 37.2 ± 9.4 years and 79.5% were male. 57.6% respondents were exposed to occupational noise while only 56.9% of them took ear protection measures. The mean BMI value of the respondents was 24.0 ± 3.7 and 69.3% was overweight (BMI ≥ 24). Sixty-three thousand one hundred sixty-one respondents (99.6% of self-reported occupational noise exposed) participated in the pure tone audiometry tests and the mean BHFTA value of them was 22.5 ± 10.8 with 25.7% had high-frequency hearing loss (BHFTA > 25dB). The mean MTMV value of better ear was 18.8 ± 6.4 with 8.6% had impairment on speech frequency hearing functions (*MTMV*_*better*_>25dB). Thirty-one thousand one hundred eighty-four respondents had ECG abnormalities and 20.6% had hypertension. Detailed descriptive characteristics of the respondents are listed in Table 1.

**Table 1:** Descriptive characteristics of wokers.

**Characteristics**	**Category/Range**	***n***	**%**	**Mean**	**SD**
Age	18–60	101,605		37.2	9.4
Height (meter)	1.40–2.00	59,085		1.7	0.1
Weight (kg)	35.0–162.0	59,096		69.0	12.8
Exposure time (year)	0–44	101,605		9.6	9.3
BMI	12.2–45.0	59,075		24.0	3.7
	< 18.5	2,876	2.8		
	18.5–23.9	28,338	27.9		
	≥24	16,726	69.3		
SBP (mmHg)	38–247	101,605		124.8	15.9
DBP (mmHg)	41–176	101,605		78.8	11.3
MTMVL	−6–116	63,163		20.3	7.4
MTMVR	−6–112	63,160		20.0	7.4
BHFTA	−15–112	63,161		22.6	10.8
Sex	Male	80,825	79.5		
	Female	20,780	20.5		
Occupational noise exposure	No	43,033	42.4		
	Yes	58,572	57.6		
Ear protection	No	64,680	63.7		
	Yes	36,925	36.3		
ECG	Normal	68,678	68.8		
	Abnormal	31,163	31.2		
Hypertension	No	80,699	79.4		
	Yes	20,906	20.6		

SD, Standard deviation; BMI, Body mass index; SDP, Systolic blood pressure; DBP, Diastolic blood pressure; BHFTA, Binaural high frequency threshold average; MTMVL, Monaural threshold of weighted value of the left ear; MTMVR, Monaural threshold of weighted value of the right ear; ECG, Electrocardiogram.

There were more male in workers exposed to occupational noise (83.6%) than other risk factors (74.1%) (*p* < 0.001). Compared to workers exposed to other risk factors, workers exposed to occupational noise were younger (37.1 ± 9.5) (*p* < 0.001), yet had a higher mean BMI (24.1 ± 3.7) (*p* < 0.001) and 30.4% were overweight or obese (48.4% in those had BMI records). Only 56.9% workers would use hearing protective equipment though they were exposed to occupational noise. Higher rate of high-frequency hearing loss (25.3%), speech frequency hearing loss (8.8%), ECG abnormalities (31.9%) and hypertension (21.0%) were found in workers exposed to occupational noise than others (3.3% high-frequency hearing loss, 0.7% speech frequency hearing loss, 30.2% ECG abnormalities and 20.0% hypertension, respectively, all *ps* < 0.001). Results of comparisons of the characteristics between workers exposed to different risk factors are listed in Table 2.

**Table 2:** Comparisons of the characteristics between workers exposed to different risk factors.

	**Other risk factors**		**Occupational noise**		***p***
	**(*****n*** = **43,033)**		**(*****n*** = **58,572)**		
	***n***	**%**	***n***	**%**	
Sex					< 0.001
Male	31,866	74.1	48,959	83.6	
Female	11,167	25.9	9,613	16.4	
Ear protection					< 0.001
No	39,440	91.7	25,240	43.1	
Yes	3,593	8.3	33,332	56.9	
BHFTA					< 0.001
≤ 25	3,405	7.9	43,552	74.4	
>25	1,403	3.3	14,801	25.3	
Not examined	38,225	88.8	219	0.4	
MTMV_**better**_					< 0.001
≤ 25	4,523	10.5	53,175	90.8	
>25	285	0.7	5,177	8.8	
Not examined	38,225	88.8	220	0.4	
ECG					< 0.001
Normal	29,246	68.0	39,432	67.3	
Abnormal	12,682	29.5	18,481	31.6	
Not examined	1,105	2.6	659	1.1	
Hypertensive					< 0.001
No	34,408	80.0	46,291	79.0	
Yes	8,625	20.0	12,281	21.0	
Family history of hypertension					< 0.001
No	41,938	97.5	54,702	93.4	
Yes	1,095	2.5	3,870	6.6	
TC					< 0.001
Normal	16,882	39.2	23,383	39.9	
Abnormal	956	2.2	1,498	2.6	
Not examined	25,195	58.6	33,691	57.5	
TBIL					< 0.001
Normal	20,834	48.4	27,344	46.7	
Abnormal	3,709	8.6	5,012	8.6	
Not examined	18,490	43.0	26,216	44.8	
Age	37.3 ± 9.3		37.1 ± 9.5		< 0.001
BMI	23.8 ± 3.6		24.1 ± 3.7		< 0.001
< 18.5	1,091	2.5	1,785	3.0	< 0.001
18.5–23.9	11,062	25.7	17,276	29.5	
24.0–26.9	6,155	14.3	10,571	18.0	
≥27	3,856	9.0	7,279	12.4	
Not examined	20,869	48.5	21,661	37.0	
Exposure time	9.4 ± 9.2		9.7 ± 9.4		< 0.001
≤ 3 years	14,674	34.1	17,176	31.0	< 0.001
4–10 years	14,154	32.9	20,775	35.5	
11–20 years	8,228	19.1	10,795	18.4	
≥21 years	5,977	13.9	8,826	15.1	
Fasting glucose	5.3 ± 1.3		5.3 ± 1.4		0.080
Normal	21,965	51.0	31,134	53.2	< 0.001
Hyperglycemia	2,310	5.4	3,550	6.1	
Hypoglycaemia	392	0.9	699	1.2	
Not examined	18,366	42.7	23,189	39.6	
ALAT	24.9 ± 23.3		27.1 ± 24.5		< 0.001
SBP	124.2 ± 16.2		125.2 ± 15.7		< 0.001
DBP	78.2 ± 11.5		79.2 ± 11.2		< 0.001

TC, Total cholesterol; TBIL, Total bilirubin; ALAT, Alanine aminotransferase.

### Results of univariate and multivariable analyses

Univariate analyses were used to identify risk factors of speech frequency hearing loss, ECG abnormalities and hypertension. According to our analysis, workers exposed to occupational noise had a statistically significant (*p* < 0.001) higher prevalence of high-frequency hearing loss (25.3%), speech frequency hearing loss (8.8%), ECG abnormalities (31.6%) and hypertension (21.0%) than those not (0.7, 29.5 and 20.0%, respectively).The prevalence of hearing loss and hypertension increased with the time of exposure to occupational hazards (*p* < 0.001). For workers exposed to occupational hazards for over 20 years, the prevalence rates of high-frequency hearing loss, speech frequency hearing loss and hypertension reached 29.0, 13.1, and 36.9%, respectively. Besides, workers who bore abnormal BHFTA or MTMV had higher rate of ECG abnormalities and hypertension. More details are shown in Table 3.

**Table 3:** Univariate analysis for the possible predictive factors of speech frequency hearing loss, ECG abnormalities and hypertension.

**Variable**	**MTMV**_**better**_			***p***	**ECG**			***p***	**Hypertensive**		***p***
	**Normal**	**Abnormal**	**Not examined**		**Normal**	**Abnormal**	**Not examined**		**No**	**Yes**	
Sex				< 0.001				< 0.001			< 0.001
Male	60.0%	5.8%	34.2%		67.7%	31.0%	1.4%		77.5%	22.5%	
Female	44.4%	3.8%	51.9%		67.3%	29.5%	3.1%		86.8%	13.2%	
Occupational noise exposure				< 0.001				< 0.001			< 0.001
No	10.5%	0.7%	88.8%		68.0%	29.5%	2.6%		80.0%	20.0%	
Yes	90.8%	8.8%	0.4%		67.3%	31.6%	1.1%		79.0%	21.0%	
BHFTA				< 0.001				< 0.001			< 0.001
Normal	98.3%	1.7%	0.0%		67.6%	31.3%	1.1%		81.3%	18.7%	
Abnormal	71.1%	28.9%	0.0%		67.1%	32.2%	0.7%		72.8%	27.2%	
Not examined	0.0%	0.0%	100.0%		67.8%	29.3%	2.9%		80.0%	20.0%	
MTMV_better_	—							< 0.001			< 0.001
Normal		67.6%	31.3%	1.1%		80.0%	20.0%	
Abnormal		66.2%	33.4%	0.3%		70.0%	30.0%	
Not examined		67.8%	29.3%	2.9%		80.0%	20.0%	
ECG				< 0.001	—						< 0.001
Normal	56.8%	5.3%	38.0%			79.4%	20.6%	
Abnormal	58.0%	5.9%	36.1%			79.0%	21.0%	
Not examined	35.6%	1.1%	63.3%			88.8%	11.2%	
Fasting glucose				< 0.001				< 0.001			< 0.001
Normal	58.9%	5.5%	35.6%		67.3%	32.3%	0.4%		81.1%	18.9%	
Hyperglycemia	53.6%	13.1%	33.3%		67.9%	31.7%	0.4%		54.6%	45.4%	
Hypoglycaemia	61.2%	4.1%	34.6%		56.7%	43.0%	0.3%		85.7%	14.3%	
Not examined	54.4%	4.2%	41.4%		68.2%	28.1%	3.7%		80.7%	19.3%	
TC				< 0.001				< 0.001			< 0.001
Normal	57.6%	7.2%	35.2%		67.4%	32.6%	0.0%		78.2%	21.8%	
Abnormal	58.0%	10.0%	32.0%		70.7%	29.2%	0.1%		62.5%	37.5%	
Not examined	56.2%	4.0%	39.9%		67.6%	29.4%	3.0%		81.0%	19.0%	
TBIL				< 0.001				< 0.001			0.004
Normal	56.8%	5.6%	37.6%		72.9%	26.6%	0.5%		79.5%	20.5%	
Abnormal	56.1%	7.3%	36.6%		71.3%	28.5%	0.2%		78.1%	21.9%	
Not examined	56.9%	4.7%	38.4%		61.1%	35.5%	3.3%		79.6%	20.4%	
Family history of hypertension				< 0.001				< 0.001			< 0.001
No	56.7%	4.6%	38.7%		67.3%	30.9%	1.8%		80.5%	19.5%	
Yes	57.8%	20.2%	22.0%		72.9%	27.1%	0.0%		58.9%	41.1%	
Family history of cardiac disease				< 0.001				0.8%			< 0.001
No	56.8%	5.3%	37.9%		67.6%	30.7%	1.7%		79.5%	20.5%	
Yes	55.8%	20.4%	23.8%		75.1%	24.9%	0.0%		61.5%	38.5%	
Age	36.5 ± 9.3	44.4 ± 8.5	37.2 ± 9.3	< 0.001	37.4 ± 9.3	36.9 ± 9.8	34.0 ± 7.9	< 0.001	35.9 ± 9.0	42.1 ± 9.4	< 0.001
BMI	24.1 ± 3.7	24.4 ± 3.5	23.8 ± 3.6	< 0.001	24.3 ± 3.6	23.5 ± 3.7	22.2 ± 3.5	< 0.001	23.4 ± 3.4	26.0 ± 3.8	< 0.001
< 18.5	63.1%	4.3%	32.6%	< 0.001	49.0%	48.4%	2.6%	< 0.001	94.5%	5.5%	< 0.001
18.5–23.9	60.3%	6.6%	33.1%		60.8%	37.9%	1.3%		86.7%	13.3%	
24.0–26.9	61.7%	8.1%	30.2%		67.7%	31.6%	0.7%		74.0%	26.0%	
≥27	64.3%	8.0%	27.8%		69.6%	29.9%	0.5%		59.3%	40.7%	
Not examined	50.1%	2.9%	47.0%		72.8%	24.5%	2.7%		80.9%	19.1%	
Exposure time	9.3 ± 9.0	15.0 ± 11.6	9.3 ± 9.1	< 0.001	10.0 ± 9.4	9.1 ± 9.2	5.2 ± 5.6	< 0.001	8.7 ± 8.6	13.3 ± 10.9	< 0.001
≤ 3 years	56.0%	3.5%	40.5%	< 0.001	64.1%	33.2%	2.7%	< 0.001	86.7%	13.3%	< 0.001
4–10 years	60.2%	3.9%	36.0%		67.8%	30.4%	1.8%		81.6%	18.4%	
11–20 years	55.5%	5.4%	39.1%		70.1%	28.8%	1.1%		75.6%	24.4%	
≥21 years	52.1%	13.1%	34.8%		71.6%	28.1%	0.3%		63.1%	36.9%	
SBP	124.9 ± 15.5	128.2 ± 17.3	124.1 ± 16.3	< 0.001	124.8 ± 15.5	125.0 ± 16.8	120.0 ± 15.8	< 0.001	119.3 ± 11.2	145.9 ± 13.9	< 0.001
DBP	79 ± 11.2	80.7 ± 11.8	78.1 ± 11.5	< 0.001	78.7 ± 11.1	78.9 ± 11.8	77.5 ± 10.8	< 0.001	75.0 ± 8.3	93.2 ± 9.6	< 0.001

**Figure 1:**
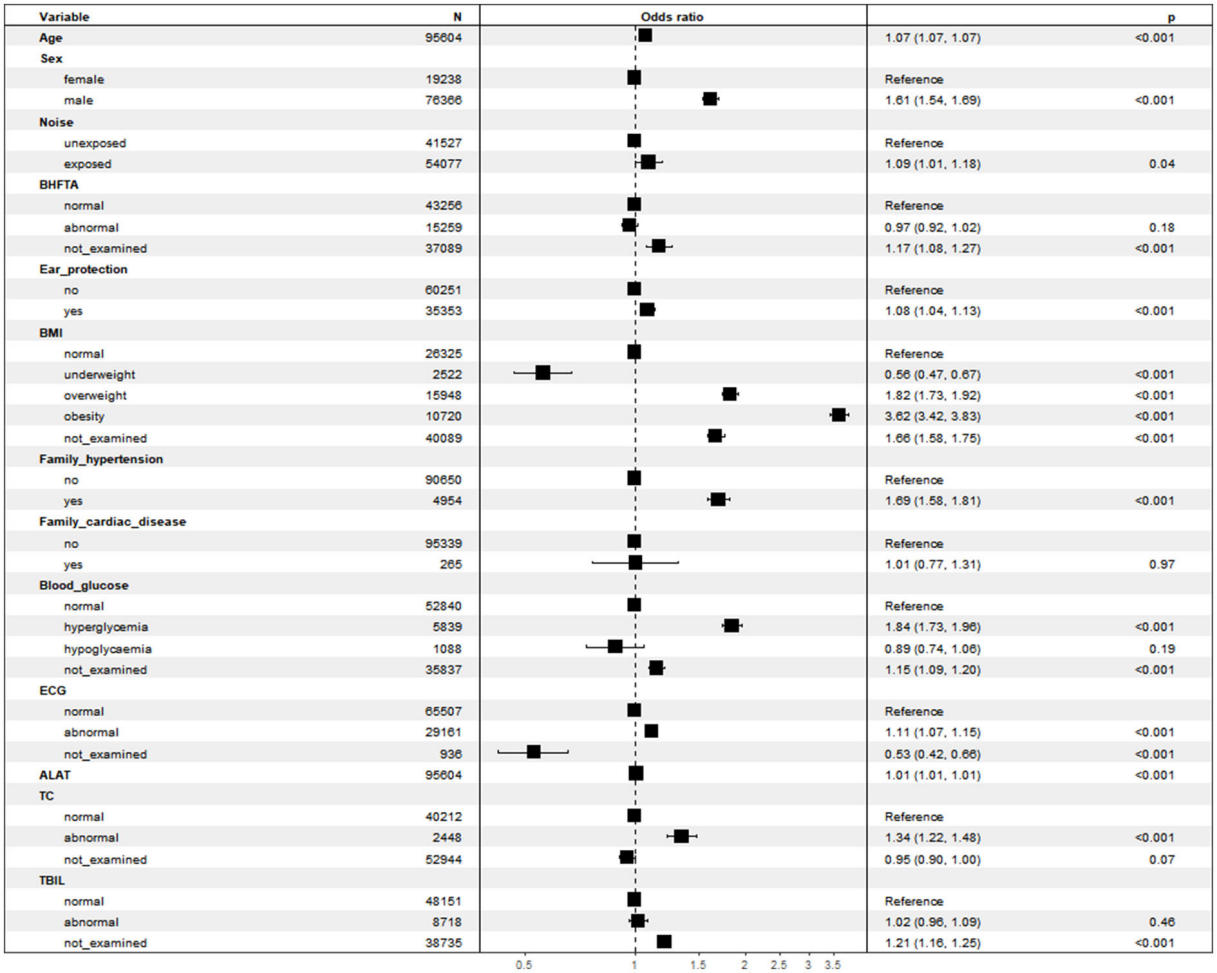
Forest plot of multivariable logistic regression.

Binary logistic regressions were performed to calculate odds ratios (ORs) for the risk of hypertension (Figure 1). Occupational noise exposure (OR = 1.09, 95% CI: 1.01–1.18, *p* = 0.04), growth of age (OR: 1.07, 95% CI: 1.07–1.07, *p* < 0.001) and BMI, hyperglycemia (OR: 1.84, 95% CI: 1.73–1.96, *p* < 0.001), hypercholesterolemia (OR = 1.34; 95% CI 1.22–1.48; *p* < 0.001), ECG abnormalities (OR = 1.11; 95% CI 1.07–1.15; *p* < 0.001) and family history of hypertension (OR = 1.69; 95% CI 1.58–1.81; *p* < 0.001) were risk factors of hypertension for workers. Male workers had a relatively higher hypertension risk than female workers (OR = 1.61; 95% CI 1.54–1.69; *p* < 0.001).

### Nomogram construction and validation

**Figure 2:**
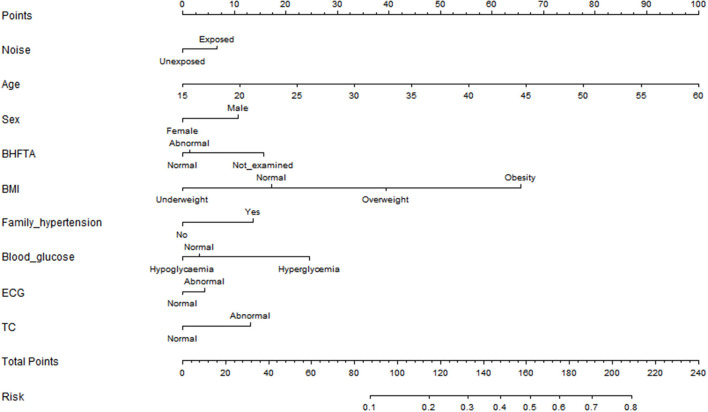
Nomogram to predict incidence risk of hypertension.

Nomograms were the visualization of statistical predictive models specifically developed to enable individualized prognosis prediction and may convey the results of a various statistical models. In our study, the intention was to predict a binary outcome of workers' hypertension (yes/no) based on the above results. After listwise deletion of participants with missing values, 37,406 workers were included in the analysis. For internal validation, the workers were randomly assigned into two groups with a ratio of 7:3 following a randomization sequence:training set (*n* = 26,004) and internal validation set (*n* = 11,402). For external validation, we used data from a survey conducted in a cigarette factory in Wuhan from July 2020 to August 2020. Eight hundred seventy-nine workers were included in this study for external validation. With the results in Figure 1, we incorporated sex, age, BMI, noise exposure, BHFTA, ECG results, TC results, family history of hypertension and blood glucose results into the nomogram to predict incidence risk of hypertension [R^2^ = 0.225, C-index = 0.760 (95% CI 0.754–0.767)] (Figure 2). The characteristics of participants in training set, internal validation set and external validation set are shown in Table 4.

**Table 4:** Characteristics of participants in training set and validation sets.

**Characteristic**	**Training set**		**Internal validation set**		***p***	**External validation set**		***p***
	**(*****n*** = **26,004)**		**(*****n*** = **11,402)**			**(*****n*** = **879)**		
	***n***	**%**	***n***	**%**		***n***	**%**	
**Sex**								
Female	5,430	20.4	2,353	20.6	0.616	199	22.6	0.107
Male	21,174	79.6	9,049	79.4		680	77.4	
Age	38.7 ± 9.5	38.6 ± 9.5	0.231	37.8 ± 10.7	0.021
**Occupational noise exposure**								
No	10,576	39.8	4,558	40	0.685	236	26.8	< 0.001
Yes	16,028	60.2	6,844	60		643	73.2	
BMI								
< 18.5	1,096	4.1	468	4.1	0.855	49	5.6	< 0.001
18.5–23.9	12,344	46.4	5,288	46.4		524	59.6	
24.0–26.9	7,828	29.4	3,396	29.8		181	20.6	
≥27	5,336	20.1	2,250	19.7		125	14.2	
BHFTA								
Normal	12,306	46.3	5,247	46	0.754	593	67.5	< 0.001
Abnormal	5,378	20.2	2,287	20.1		149	16.9	
Not examined	8,920	33.5	3,868	33.9		137	15.6	
**Exposure time**								
≤ 3 years	5,289	19.9	2,267	19.9	0.706	184	24.3	< 0.001
4–10 years	8,279	31.1	3,582	31.4		200	26.4	
11–20 years	6,345	23.8	2,748	24.1		139	18.4	
≥21 years	6,691	25.2	2,805	24.6		234	30.9	
**Family history of hypertension**								
No	23,475	88.2	10,095	88.5	0.406	568	64.6	< 0.001
Yes	3,129	11.8	1,307	11.5		311	35.4	
**ECG**								
Normal	17,707	66.6	7,597	66.6	0.893	667	75.9	< 0.001
Abnormal	8,897	33.4	3,805	33.4		212	24.1	
**Total cholesterol (TC)**								
Normal	25,047	94.1	10,734	94.1	0.981	845	96.1	0.013
Abnormal	1,557	5.9	668	5.9		34	3.9	
**Fasting glucose**								
Normal	23,159	87.1	9,891	86.7	0.624	800	91	< 0.001
Hyperglycemia	2,858	10.7	1,263	11.1		78	8.9	
Hypoglycaemia	587	2.2	248	2.2		1	0.1	
**Hypertensive**								
No	20,376	76.6	8,725	76.5	0.885	785	89.3	< 0.001
Yes	6,228	23.4	2,677	23.5		94	10.7	

**Figure 3:**
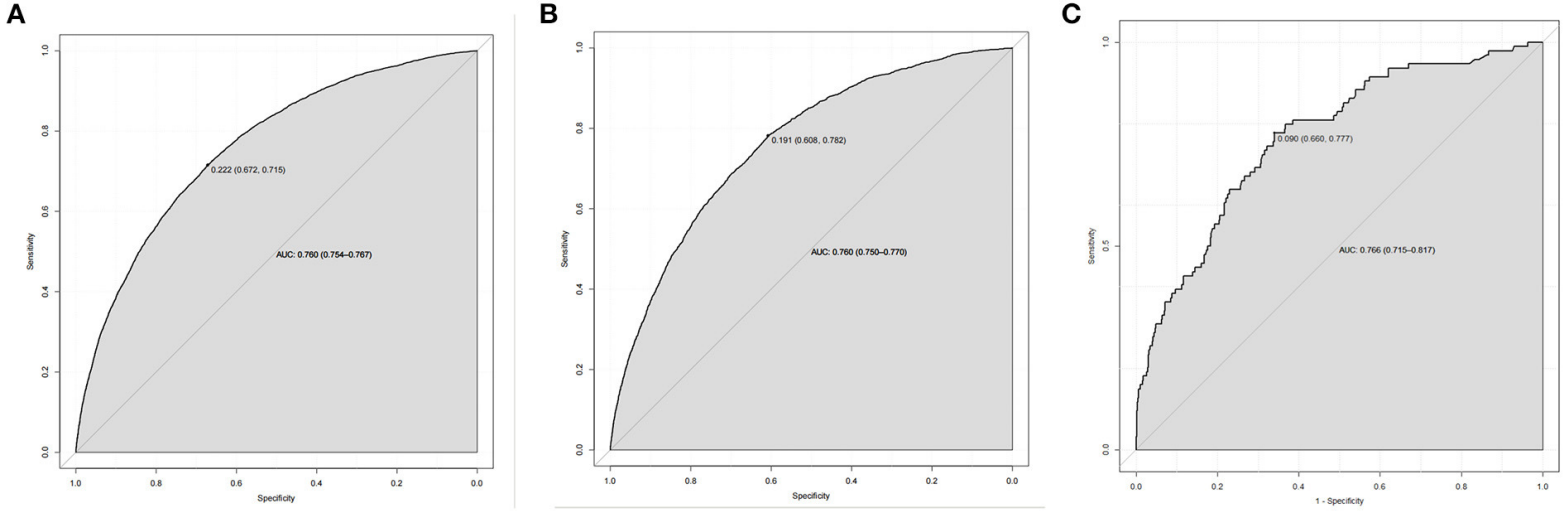
ROC curves. **(A)** Training set. **(B)** Internal validation set. **(C)** External validation set. ROC, receiver operating characteristic; AUC, area under the ROC curve.

**Figure 4:**
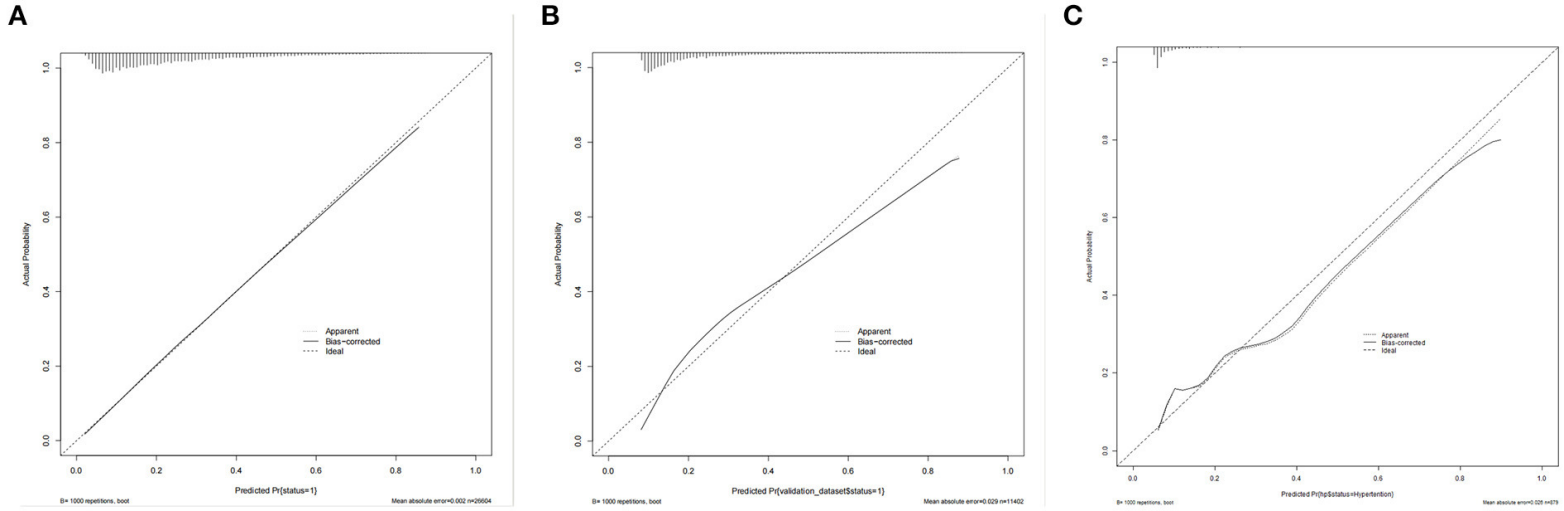
Calibration curve for predicting probability of hypertension. **(A)** Training set. **(B)** Internal validation set. **(C)** External validation set.

**Figure 5:**
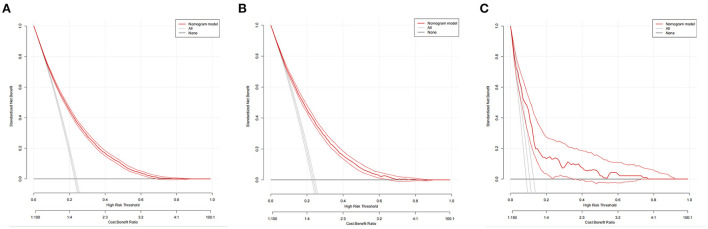
Decision curve analysis in prediction of hypertension. **(A)** Training set. **(B)** Internal validation set. **(C)** External validation set.

The nomogram had good accuracy in training set (AUC, 0.760; 95% CI 0.754–0.767) (Figure 3A), internal validation set (AUC, 0.760; 95% CI 0.750–0.770) (Figure 3B) and external validation set (AUC, 0.766; 95% CI 0.715–0.817) (Figure 3C). The calibration curves were close to the ideal diagonal line and showed good calibration in training set (Figure 4A), internal validation set (Figure 4B) and external validation set (Figure 4C). Moreover, the DCA showed significant net benefit of the predictive model (Figure 5A), as well as that in the validation cohorts (Figures 5B,C). These data demonstrated that our nomogram had a high potential for clinical utility.

### Website of nomogram

To facilitate use of our prediction model, a dynamic nomogram was established publicly available online (https://hbpdynnomo.shinyapps.io/DynNom_HBP_workers/) using the DynNom package of R. It may dynamically predict the hypertension risk of workers on the website in a user-friendly way.

## Discussion

Based on occupational health examination data, this study analyzed the current prevalence of hearing loss, ECG abnormalities and hypertension among workers with different occupational risks exposure in Wuhan. We further explored the risk factors of workers' hypertension. According to our study, occupational noise was the most exposed occupational hazard factor for workers in Wuhan and 57.6% respondents were exposed to it. 31.2% workers showed ECG abnormalities and 20.6% had hypertension. Such a high frequency of occupational noise exposure suggests that importance should be placed on monitoring the hazards of occupational noise and targeted occupational protection measures should be taken as early as possible. Among respondents participated in pure tone audiometry tests, 25.7% had high-frequency hearing loss and 8.6% had speech frequency hearing loss. Workers exposed to occupational noise had higher rate of high-frequency hearing loss (25.3%), speech frequency hearing loss (8.8%), ECG abnormalities (31.6%) and hypertension (21.0%), reconfirming the negative effect of occupational noise on auditory system and cardiovascular system (1, 14, 38).

Studies have suggested that acoustic overstimulation may contribute to the pathogenesis and biochemical changes that result in hearing loss (39, 40). The continued and evolving research involving noise-induced hearing loss (NIHL) has determined that there is a close relationship between the occurrence of NIHL and changes in some genes, cell metabolism, cell apoptosis and so forth (41). Thus, workers exposed to occupational noise may have heightened risk for hearing loss. Damaging effects of occupational noise on cardiovascular system have been presented in previous studies (17, 19, 20) as well. Several mechanisms through which noise contribute to cardiovascular system impairment are proposed: changes to gene net-works, epigenetic pathways, the gut microbiota, circadian rhythm, neuronal excitability and signal transduction, oxidative stress, inflammation and metabolism (13). Experimental evidence shows that noise may cause an increase in stress hormone release and in circulating angiotensin II (Ang II) levels with significant stress-induced increase in blood pressure (38). The effects of noise exposure on cognitive function (18), mental health (42) and spiritual wellbeing (43) may also play an important role in the process of occupational noise exposure affecting cardiovascular health. Surprisingly, we found that protective measures such as wearing ear muffs and earplugs could not reduce the risk of hypertension in workers. One possibility is that protective measures of the workers are not in place. Workers do not wear or do not wear ear protection equipment correctly, or the protective effect of ear protection equipment is limited. Common occupational ear protection articles are more used to protect workers against high-frequency noise, while protection against low-frequency noise is limited. Alternatively, the influence of occupational noise on hypertension has nothing to do with ear protection and more valid and relevant measures should be taken. Effects of wearing protective equipment and impacts of occupational noise at different frequencies and intensities on the cardiovascular system of workers deserve further exploration. Besides, we found a sex-dependent effect of noise on hypertension, which is consistent with the previous study (44). It may be related with the fact that the risk pattern for hypertension (45) and nature of the work (46) are different for male and female.

Although univariate analysis showed that workers with hearing loss had greater prevalence of hypertension, multivariate analysis showed that the relationships between the two were not significant. In our study, workers exposed to occupational noise (OR = 1.09; 95% CI 1.01–1.18; *p* = 0.04) faced higher risk of hypertension than those not and the association between hypertension and occupational noise exposure was confirmed existed regardless whether the worker had hearing loss. A systematic review suggested that exposure to noise at work was consistently positively associated with hypertension [Hazard ratio (HR) = 1.68; 95% CI 1.10–2.57] (26). Previous research suggests that noise could raise blood pressure without impairing auditory sensitivity (24). However, the healthy worker survivor effect (47) should not be ignored as well. Aside from the muting effects of hearing loss, the healthy worker survivor effect would also conceal the impacts of occupational noise on blood pressure. In addition, the muting correlation between hearing loss and hypertension may also be related to their positive correlations with age. Hyperglycemia (OR = 1.84; 95% CI 1.73–1.96; *p* < 0.001), hypercholesterolemia (OR = 1.34; 95% CI 1.22–1.48; *p* < 0.001) and ECG abnormalities (OR = 1.11; 95% CI 1.07–1.15; *p* < 0.001) were significant predictors as well. It should be noted that hypertension, dyslipidemia and dysglycemia are risk factors of CVD (48) and hypertension combined with dysglycemia may greatly exacerbate the risk of CVD (49).

We provided a visualization of workers' hypertension prediction using nomogram based on binary logistic regression analysis. Available sociodemographic characteristics and clinical parameters were used in the nomograms, which was convenient and quick for screening the high-risk individuals. Previous nomogram suggests that age, sex, early life factors, family history of the disease, and lifestyle factors may predict the risk of hypertension (44, 50). Given the research subjects of this study are workers exposed to occupational hazards and data limit, this study finally included age, sex, occupational noise exposure status and physical examination results. Results revealed that sex, age, BMI, occupational noise exposure status, ECG results, TC results, family history of hypertension and blood glucose results were predictors of hypertension in workers with different occupational hazards in Wuhan. Our nomogram showed good predictive accuracy and validity. It may help identify high-risk workers and facilitate timely, effective and targeted prevention interventions so as to improve occupational health.

There are several limitations in our study as follows. First, occupational health examination data are by their nature observational studies, where data tend to be collected by clinicians rather than investigators. Data used for this study were cross-sectional and we can only demonstrate associations rather than prove causality, nor did we verify the mechanism. Secondly, we only studied whether exposure to occupational noise had an impact on the risk of hypertension, but did not specifically estimate the impact of noise at different frequencies and intensities on health. Last but not least, despite adjusting some factors in the present study, there are still some confounders that may influence the results not included, which should be considered in subsequent studies.

## Conclusion

Occupational noise exposure of workers may elevate their hypertension risk. Standard ear protection measures should be strengthened and more effective and relevant hypertension prevention measures should be taken. Our nomogram may help identify high-risk workers and facilitate timely interventions so as to improve occupational health.

## Data availability statement

The raw data supporting the conclusions of this article will be made available by the authors, without undue reservation.

## Ethics statement

Written informed consent was obtained from the individual(s) for the publication of any potentially identifiable images or data included in this article.

## Author contributions

LZ and XT designed the study. LZ, ZC, WY, WF, FH, ZP, and GY collected the data. LZ performed statistical analysis. LZ, SC, and ZC drafted this manuscript. LZ, XT, and GY revised the manuscript. XT and GY had primary responsibility for final content. All authors were involved in the revisions and approved the final version of the manuscript.
